# Survival, remission, and quality of life in diabetic cats

**DOI:** 10.1111/jvim.16625

**Published:** 2023-01-13

**Authors:** Ninni Rothlin‐Zachrisson, Malin Öhlund, Helena Röcklinsberg, Bodil Ström Holst

**Affiliations:** ^1^ Department of Clinical Sciences Swedish University of Agricultural Sciences Uppsala Sweden; ^2^ Swedish Medical Products Agency Uppsala Sweden; ^3^ Department of Animal Environment and Health Swedish University of Agricultural Sciences Uppsala Sweden

**Keywords:** feline, outcome, owner, perceptions, treatment, wet diet

## Abstract

**Background:**

Remission is documented in a substantial proportion of cats with diabetes. The effects of diabetes mellitus (DM) on the lives of cats and their owners should be considered when evaluating treatment success.

**Objectives:**

To study outcome in cats with DM and the impact DM has on the life situation of cat and owner.

**Animals:**

Domestic and pedigree cats with a diagnosis of DM (n = 477) insured by a Swedish insurance company during 2009 to 2013.

**Methods:**

Retrospective cross‐sectional study. A questionnaire was sent to 1369 owners of cats diagnosed with DM. The questions concerned the cat, treatment, owner perceptions of the disease and treatment and disease outcome. Data were analyzed using multiple linear and logistic regression, with outcomes set as survival for more than 4 weeks after diagnosis, survival time, achieving remission, remission without relapse and quality of life (QoL) for the cat.

**Results:**

The response rate was 35%, leaving 477 questionnaires for analysis. The remission rate among treated cats was 29% (118/405). Feeding a commercially available wet diet was associated with both remission (OR 3.16, 95% confidence interval 1.27‐8.12) and remission without relapse (OR 14.8, 95% confidence interval 2.25‐153.8). Remission was associated with a better QoL for the cat.

**Conclusions and Clinical Importance:**

The association between feeding a commercially available wet diet and remission is important and strengthens the role of diet in treatment of DM in cats. Linking remission and a better QoL for the cat emphasizes remission as a goal in disease management.

**Abbreviations**
CA: commercially available
CI: confidence interval
DM: diabetes mellitus
HBGM: home blood glucose monitoring
LC: low carbohydrate
OR: odds ratio
QoL: quality of life

## 1 INTRODUCTION

The quality of life of chronically sick animals and their owners is attracting increased attention, and its importance for owners and clinicians is substantial.^1^, ^2^, ^3^, ^4^, ^5^, ^6^, ^7^ Diabetes mellitus (DM) is a common chronic endocrine disease in cats.^8^, ^9^, ^10^, ^11^ Caring for a diabetic cat entails potentially adversely effects on owners' and cats' lifestyle and quality of life (QoL). Long time management of DM includes minimization of clinical signs, and diabetic remission is documented in a substantial proportion of cats.

Associations between treatment regimen and remission have been investigated,^12^, ^13^, ^14^, ^15^, ^16^, ^17^, ^18^, ^19^, ^20^ but because of shortage of well‐designed studies no clear evidence of a superior treatment protocol exists.^21^ Adjustments toward an ultralow carbohydrate diet are widely considered optimal for disease control,^22^, ^23^, ^24^ although only a few veterinary trials have compared low carbohydrate diets to others.^18^, ^19^, ^20^

Despite DM having a fairly good prognosis, it has been estimated that 1 out of 10 owners choose euthanasia upon DM diagnosis^1^ and 10% to 17% of cats are euthanized within a few weeks of diagnosis.^25^, ^26^, ^27^ Alongside glycemic control, the psychological and social effects of DM and its treatment on both owner and cat should be considered when evaluating treatment success.^2^ Owners' perceptions of disease management and concern for the wellbeing of their diabetic cat might influence their choice of treatment or euthanasia. These perceptions are thus crucial for the owners' commitment to and care for the diabetic cat, and hence common sources of owner distress need to be recognized by the treating veterinarian for successful management of DM. Studies reveal that feeling tied up and worrying about costs related to treatment are commonly reported issues among owners of diabetic cats,^1^, ^2^, ^3^, ^28^ whereas initial concerns and negative impact associated with treatment seem to decrease significantly with time.^3^, ^29^

Answers from a questionnaire sent to owners of cats diagnosed with DM were analyzed to investigate associations between treatment regime and owners' perceptions of outcome, including QoL, for the cat. The aim of this study was to study disease outcome in cats diagnosed with DM and the impact DM has on the life situation of the cat and its owner.

## 2 MATERIALS AND METHODS

### 2.1 Study sample and questionnaire

A questionnaire was sent to all owners of cats insured by Agria Pet Insurance with a DM diagnosis during 2009 to 2013 (n = 1369). The cats were identified based on 4 diagnostic codes (DM, DM without complication, DM with complication and DM with ketoacidosis). The cat owners were recruited to the study by e‐mail with a web link to the questionnaire.

The questionnaire was available during a 4‐month period through an online provider (Netigate) and consisted of 46 questions. Some of the results—those relating to environmental risk factors for DM in cats—have been published.^30^ The answers to 37 questions were analyzed in the present study. To confirm case status, all respondents had to give a positive answer to the question “Has your cat ever been diagnosed with diabetes? The diagnosis must have been made by a veterinarian. Diabetes means that the cat's blood sugar is elevated for a longer period of time.” To confirm cases of diabetic remission, the respondents had to give a positive answer to the question “Has your cat recovered from its diabetes? Recovery meaning a normalized blood sugar and the cat no longer needing medication (insulin or oral tablets).” For a summary of the questions, see Table 1. For detailed information about the questions, see Appendix S1.

**TABLE 1:** Overview of the questions asked to owners of diabetic cats and the answer options, including answer grouping for data analysis (n = 477)

Questions	Answer options and grouping of answers for data analysis (all questions included an “Other/Decline to answer” alternative and a free text section)
Cat characteristics and survival time	
Breed	Domestic, Burmese, Norwegian Forest Cat, Exotic/Persian, Other pedigree^a^
Year of birth	For example, 2002
Sex	Male, Neutered male, Female, Neutered female
Confinement	Indoor, Outdoor^b^
Multicat household	Yes, No^c^
Cat alive at time of survey?	Yes, No
If not alive, what year did your cat die?	For example, 2006
Main reason for death/euthanasia	Trauma/accident, Age, Disease
If disease, what kind of disease?^d^	Airways, Circulatory system, Diabetes or other endocrine, Gastrointestinal, Oral cavity, Orthopedic or neurological, Tumor, Urinary system
Cat health through life (before diabetes diagnosis)	Never/rarely sick or hurt, Occasionally sick or hurt, Often sick/recurrent problems/chronic disease
If often sick/recurrent problems/chronic disease, what systems were affected?^d^	Airways, Circulatory system, Endocrine, Gastrointestinal, Infectious, Oral cavity, Orthopedic or neurological, Reproductive, Skin, Tumor, Urinary system
Vaccination status	Yearly doses, Every other year, Occasionally, As a kitten, Not vaccinated
Treatment with progestins or corticosteroids the year before diabetes diagnosis	Yes, corticosteroids, Yes, progestins, No^e^
Body condition score of your cat the year before diabetes diagnosis^f^	1‐5
Owner and household	
Year of birth	Decade of birth, for example, 1960‐69
Sex	Male, Female
Number of adults in household	1, 2, 3 or more
Children <18 years old in household	Yes, No
Place of residence	City (>200 000 inhabitants), Town (200‐200 000 inhabitants), Countryside
Diabetes diagnosis, initial survival and treatment	
What year did your cat contract diabetes? (When clinical signs were first seen or, if no clinical signs, when your cat received its diagnosis)	For example, 2013
What happened to your cat after diagnosis?	Euthanized/died within 4 weeks, Survived for more than 4 weeks
Reasons for euthanasia^d^	The cat did not survive despite initiated treatment, Presence of other diseases, The treatment did not work, Treatment was too difficult for me/my family, Wanted to limit the suffering for the cat, The treatment was too expensive, The cat did not receive any treatment, Experienced poor support from veterinarian
What treatment did your cat receive?^d^	Insulin^g^, Dietary adjustments, Hypoglycemic tablets (eg, glipizide), Admitted to hospital for care, No particular treatment
Change of diet upon diabetes diagnosis	Yes, Yes, partly, No
What diet has your cat predominantly eaten since diagnosis? (“predominantly” meaning ≥75% of one diet; if approximately 50% of two diets, two alternatives could be chosen)^d^	Veterinary diabetes prescription dry diet (eg, Hill's m/d, Purina DM, Royal Canin Diabetic), Veterinary diabetes prescription wet diet (eg, Hill's m/d, Purina DM, Royal Canin Diabetic), Veterinary weight loss/obesity prescription diet (eg, Hills r/d or w/d), Commercially available wet food^h^
Did you practice blood glucose monitoring at home?^d^	Yes, with blood samples, Yes, with urine sticks, No
Remission	
Has your cat “recovered” from diabetes? (meaning a normal blood glucose without insulin treatment)	Yes, Yes, with relapse^i^, No
If Yes or Yes with relapse: Time from diagnosis until “recovery”	0‐3 months, 4‐6 months, 7‐12 months, 1 year or longer
Owner perceptions	
How were you affected by having a cat with diabetes?^d^	Worried about the cat's medication, Worried about complications (eg, hypoglycemia), Difficult to administer insulin, Difficult to perform blood sampling, Worried about hurting the cat during medication or blood sampling, Perceiving limitations in life due to cat's diabetes (eg, difficulties traveling), Worried about limitations to the cat's life due to diabetes, Worried about costs, Experienced expectations from others to start treatment, Experienced expectations from others to euthanize, I have not been affected by my cat's diabetes diagnosis
Describe your attitude to your cat	My cat is everything to me, My cat means a lot to me, My cat is quite important to me, My cat is not that important to me
Did you experience any conflicts in your family regarding the care of your cat?	Yes, often, Yes, sometimes, No, we agree, No, I make the decisions myself
How was the relationship with your cat affected by diabetes?^d^	I have developed a stronger bond with my cat, My cat has developed a stronger bond with me, Relationship as before, I have developed a weaker bond with my cat, My cat has developed a weaker bond with me
Quality of life (QoL)	
How has the QoL of your cat been affected by diabetes, in general, compared to before the cat got sick?	Better, no change/as before, worse^i^
Assessment of QoL of your cat at time of survey/last time alive	Excellent, good, less good, bad

^a^ <8 individuals per breed: Abyssinian, Bengal, Birman, British Shorthair, Cornish Rex, Devon Rex, European Shorthair, Maine Coon, Ocicat, Oriental Shorthair, Ragdoll, Russian Blue, Siamese, Siberian incl. Neva Masquerade, Somali, Sphynx, Other pedigree; mixed breed cats were grouped as domestic cats.

^b^ Indoor = Indoor only, Indoor with access to balcony/play pen/leash walks. Outdoor = Indoor with access to outdoors for part of the year, Outdoor and indoor, Outdoor only.

^c^ Yes = Two to three cats, Four to eight cats, Nine or more cats.

^d^ Multiple answers possible.

^e^ Yes, corticosteroids = per oral tablets or injection. Yes, progestins = per oral tablets or injection.

^f^ Assessment template of BCS provided.

^g^ Insulin = insulin injection once and/or twice day.

^h^ Other = diets or combinations of diets representing the choice of fewer than 15 (5%) owners, including commercially available dry food and Low carbohydrate dry.

^i^ Dichotomized before logistic regression analysis: Better compared to before the disease or No change/as before = Positively affected/unaffected QoL, Worse compared to before the disease = Negatively affected QoL.

### 2.2 Data analysis

In the case of missing answers for any of the variables of interest, the cat was excluded. In the case of conflicting answers, the respondent was contacted if possible and the answers were then corrected or excluded accordingly. See Table 1 for an overview of the questionnaire and, where relevant, how the answers were grouped for data analysis.

As no specific dates were requested, the cat's age at diagnosis and age at death were set to 1 January of each year. Initial survival was defined as surviving for more than 4 weeks after diagnosis. Survival time was estimated as from 31 December of the year of diagnosis to 1 January of the year of the cat's death. For a cat that was diagnosed in 2010 and died in 2012, the survival time was thus set to a minimum of 1 year.

In the data analysis for all cats, outcomes were initial survival (meaning surviving for more than 4 weeks after diagnosis) and a QoL not negatively affected by DM. For cats still alive at 4 weeks after diagnosis, disease outcome (survival time and achieving remission, with or without diabetic relapse) was analyzed.

Data analysis was performed using R.^31^ For summary statistics, frequency distribution (histogram) was used to check for normality. Mean and SD (±) were used when normality was assumed, and median and inter‐quartile range (IQR) when this was not the case. The main outcome variables were survival time, remission, and quality of life. Five different regression models were used, with outcomes set as initial survival (more than 4 weeks after diagnosis), survival time, remission, remission without relapse and quality of life for the cat not being negatively affected by DM. Univariate logistic or linear regression were used for selection of variables, including variables with *P* < .2 in further analysis. To estimate and investigate the direction and strength of the associations, multiple logistic regression analysis and linear regression were used. The final regression model was decided with a backwards elimination process combined with a lowered Akaike information criterion (AIC), and variables with *P* > .05 were excluded. In the logistic regression, analysis odds ratio (OR) was calculated with a 95% confidence interval (CI). In linear regression analysis, a 95% CI with a significance level of 5% was used. Quantile‐quantile plots (QQ‐plots) were used to assess the regression residuals for normality. Biologically plausible interactions were included and possible confounders were controlled in each regression model. Significant interactions were interpreted separately and investigated using interaction plots.

## 3 RESULTS

In total, 484/1369 (35%) complete questionnaires were received and 7 were excluded, leaving 477 questionnaires for analysis.

### 3.1 Cats and owners

Mean cat age at diagnosis was 10.7 years (±3.1). Almost 100% of the cats were neutered (99.9%), of which 71% were males and 29% were females, and most cats were not pedigrees (77%). For general information on the cats, see Table 2. The majority of the cats (333/477, 70%) were not alive at the time when their owners answered the questionnaire.

**TABLE 2:** General information on cat characteristics, disease outcome, and treatment for cats diagnosed with diabetes mellitus (n = 477)

				Remission			
General information	All cases (n = 477)	Survival ≤4 weeks (n = 72)	Survival >4 weeks (n = 405)	All (n = 118)	Relapse (n = 45)	No relapse (n = 73)	No remission (n = 287)
Breed							
Domestic	365 (76%)	46 (64%)	319 (79%)	83 (70%)	29 (64%)	54 (74%)	236 (82%)
Burmese	18 (4%)	4 (6%)	14 (3%)	5 (4%)	2 (4%)	3 (4%)	9 (3%)
Norwegian Forest Cat	38 (8%)	6 (8%)	32 (8%)	15 (13%)	6 (13%)	9 (12%)	17 (6%)
Exotic/Persian	8 (2%)	2 (3%)	6 (1%)	2 (2%)	1 (2%)	1 (1%)	4 (1%)
Other purebred	48 (10%)	14 (19%)	34 (8%)	13 (11%)	7 (16%)	6 (8%)	21 (7%)
Sex							
Neutered male	337 (71%)	52 (72%)	285 (70%)	88 (75%)	37 (82%)	51 (70%)	197 (69%)
Neutered female	137 (29%)	20 (28%)	117 (29%)	29 (25%)	8 (18%)	21 (29%)	88 (31%)
Intact female	3 (0%)	0	3 (1%)	1 (0%)	0	1 (1%)	2 (1%)
Mean age at diagnosis, years (SD)	10.7 (±3.1)	11.6 (±3.6)	10.6 (±3)	10.6 (±3)	10.6 (±3.6)	10.6 (±2.6)	10.5 (±2.9)
Presence of other chronic disease	35 (7%)	8 (11%)	27 (7%)	10 (8%)	5 (11%)	5 (7%)	17 (6%)
Indoor confinement	258 (54%)	41 (57%)	217 (54%)	76 (64%)	28 (62%)	48 (66%)	141 (49%)
Outdoor confinement	217 (45%)	31 (43%)	186 (46%)	42 (36%)	17 (38%)	25 (34%)	144 (50%)
Presence of other animals in household	321 (67%)	52 (72%)	269 (66%)	87 (74%)	34 (76%)	53 (73%)	182 (63%)
Treatment							
Insulin			360 (89%)	104 (88%)	41 (91%)	63 (86%)	256 (89%)
Dietary adjustments			378 (93%)	116 (98%)	44 (98%)	72 (99%)	262 (91%)
Oral hypoglycemics			27 (7%)	5 (4%)	2 (4%)	3 (4%)	22 (8%)
Home blood glucose monitoring							
Blood glucose			208 (51%)	70 (59%)	33 (73%)	37 (51%)	138 (48%)
Urine glucose			74 (18%)	22 (19%)	12 (27%)	10 (14%)	52 (18%)
None			147 (36%)	34 (29%)	7 (16%)	27 (37%)	113 (39%)
Diet							
DM prescription diet, dry			124 (31%)	33 (28%)	15 (33%)	18 (25%)	91 (32%)
DM prescription diet, dry and wet			60 (15%)	14 (12%)	8 (18%)	6 (8%)	46 (16%)
CA wet food and DM prescription, dry			48 (12%)	14 (12%)	7 (16%)	7 (10%)	34 (12%)
CA wet food			44 (11%)	22 (19%)	2 (4%)	20 (27%)	22 (8%)
Weight management prescription diet			27 (7%)	10 (8%)	1 (2%)	9 (12%)	17 (6%)
Other^a^			102 (25%)	25 (21%)	12 (27%)	13 (18%)	77 (27%)

Abbreviations: CA, commercially available; DM, diabetes mellitus.

a Other = diets or combinations of diets representing the choice of fewer than 15 (5%) owners, including commercially available dry food and low carbohydrate dry food.

The majority (85%) of the cat owners responding were females, and the owners' median age at cat diagnosis was 46 to 55 years (range 16‐90 years).

### 3.2 Treatment

Of the 477 cats, 405 were alive 4 weeks after diagnosis. For general information on treatment and disease outcome, see Table 2. Dietary adjustments were made for 93% of the cats and 89% were treated with insulin. Among the owners of cats receiving insulin, 181 (50%) practiced home blood glucose monitoring, 44 (12%) measured urine glucose and 23 (6%) practiced both, while 112 (31%) did not monitor blood or urine glucose. Of the 27 cats (7%) that received oral hypoglycemics, 26 also had dietary adjustments. Nine of the cats treated with oral hypoglycemic agents also received insulin at some point.

The most commonly fed diet was dry veterinary prescription diet for treatment of DM (253/405, 62%), either as a predominant diet or combined with other diets (see Table 2). Of the 405 cats, 11% were predominantly fed a CA wet diet, generally meaning a low carbohydrate diet (as obtained from free text answers).

### 3.3 Owners' perceptions and cat‐human relationship

Worries and difficulties associated with treatment of the diabetic cat are described in Table 3. The most frequently reported concern was limits to daily life, for example, difficulties traveling and finding a cat sitter. Worrying about complications involving treatment or DM itself (eg, hypoglycemia) was most common among owners of cats that had not achieved remission, as were concerns about DM entailing limits to the cat's daily life. Only 1% of owners of cats in remission without relapse expressed concern about DM limiting their cat's life. Owners of cats in remission without relapse were less worried than owners of cats with relapse or cats that had not achieved remission.

**TABLE 3:** Owner‐perceived difficulties and worries about treatment and monitoring their cat's diabetes mellitus (n = 405, multiple answers possible)

		Remission			
	All treated cats (n = 405)	All (n = 118)	Relapse (n = 45)	No relapse (n = 73)	No remission (n = 287)
Owner feeling limited	208 (51%)	55 (47%)	24 (53%)	31 (42%)	153 (53%)
Worry about complications	182 (45%)	41 (35%)	17 (38%)	24 (33%)	141 (49%)
Worry about cat's medication	117 (29%)	27 (23%)	10 (22%)	17 (23%)	90 (31%)
Difficulty measuring blood glucose	84 (21%)	25 (21%)	7 (16%)	18 (25%)	59 (21%)
Worry about costs	61 (15%)	17 (14%)	7 (16%)	10 (14%)	44 (15%)
Worry about limits to cat's life	54 (13%)	4 (3%)	3 (7%)	1 (1%)	50 (17%)
Worry about hurting the cat	40 (10%)	12 (10%)	6 (13%)	6 (8%)	28 (10%)
Difficulty injecting insulin	25 (6%)	8 (7%)	2 (4%)	6 (8%)	17 (6%)
No perceived worries or difficulties	79 (20%)	32 (27%)	8 (18%)	24 (33%)	47 (16%)

Of the 84 owners experiencing difficulties measuring blood glucose, 29 did not perform HBGM.

Of all owners, 54% (258/477) answered that their cat meant a lot to them, 41% (197/477) said that their cat was everything to them, and 3% (15/477) deemed the cat to be “quite important.” There were no associations between owners' attitudes toward their cats and the 5 different outcomes analyzed using univariable logistic regression analysis.

More than half of the owners (58%, 277/477) said that their relationship with their cat had been strengthened after the DM diagnosis, while about a third (38%, 180/477) deemed the relationship to be unchanged. Only 1% (5/477) of owners said the relationship had been worsened.

### 3.4 Outcomes for cats with diabetes mellitus diagnosis

#### 3.4.1 Survival for more than 4 weeks

**FIGURE 1:**
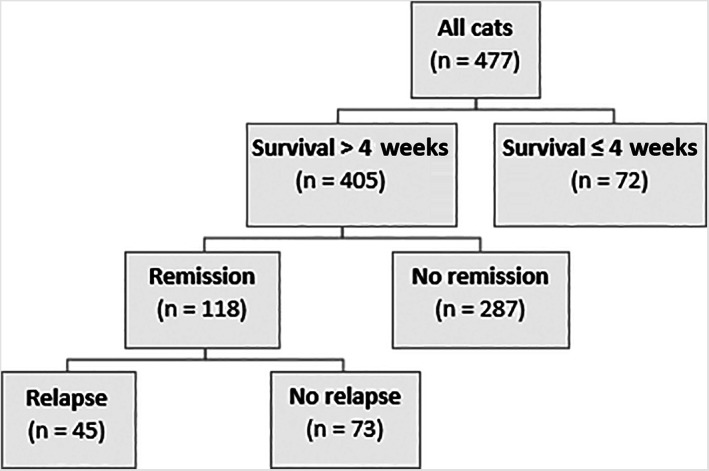
Overview of different outcomes for the studied population of cats with diabetes mellitus (n = 477)

Of the 477 cats, 405 (85%) were still alive 4 weeks or more after diagnosis, whereas 72/477 (15%) had been euthanized—see Figure 1. The most common reason for euthanasia was that the owners did not want their cats to suffer (53%) or that the prognosis was poor (32%). Other reasons for euthanasia were concurrent disease (21%), treatment being too difficult (13%) or poor support from the veterinarian (4%).

In the multiple logistic regression model, factors remaining significant for surviving for more than 4 weeks were cat age at diagnosis, owner experiencing limitations in their lifestyle, expectations from others to euthanize the cat, owner worrying about costs and the effect of DM on the cat's QoL, alongside 2 interactions: cat age at diagnosis and worrying about costs, and cat age at diagnosis and expectation to euthanize—see Table 4.

**TABLE 4:** Multiple logistic regression analysis of factors associated with different outcomes in cats with diabetes mellitus (n = 477)

Outcome and variables	OR	CI 95% (OR)		*P*
Survival for more than 4 weeks				
Worries about limitations in owner's life vs no limitations in owner's life	13.6	4.85	45.7	<.001
Worries about complications vs no worries about complications	3.00	1.33	7.11	.01
QoL better or same as before vs QoL worse	23.8	8.16	89.5	<.001
Cat age				
When worrying about costs	1.52	0.98	2.3	.05
When experiencing expectation to euthanize	0.33	0.11	0.64	.01
When no worries about costs or not experiencing expectations to euthanize	−0.14	0.76	0.99	.04
Remission				
CA wet diet vs veterinary prescription DM diet wet and/or dry	3.16	1.27	8.12	.01
Breed: Norwegian Forest Cat vs domestic cat	2.92	1.27	6.76	.01
QoL better or same as before vs QoL worse	5.25	2.86	10.3	<.001
Remission without relapse				
CA wet diet vs veterinary prescription DM diet wet and/or dry	14.8	2.25	153.8	.01
QoL better or same as before vs QoL worse	31.4	5.1	646	.002
QoL better or same				
Insulin treatment vs no insulin treatment	5.6	2.4	14	<.001
Remission without relapse vs no remission	53.9	10.6	994.6	<.001
Worries about medication vs no worries about medication	0.31	0.17	0.54	<.001
Worries about limitations in cat's life vs no worries about limitations	0.07	0.03	0.16	<.001

Abbreviations: CA, commercially available; CI, confidence interval; OR, odds ratio; QoL, quality of life.

For every additional year of the cat's age at diagnosis, the odds of surviving for more than 4 weeks decreased by 0.13. If the owner experienced expectations from others to euthanize the cat, the odds of surviving for more than 4 weeks decreased by a third (OR 0.33) with every year. This negative effect of expectations of euthanasia on initial survival was most pronounced in cats over approximately 15 years of age. In cats whose owners were worried about costs related to the cat's DM, the odds of surviving were lower in younger cats and were most pronounced in cats younger than approximately 10 years, and the odds of initial survival increased with every additional year of the cat's age (OR 1.52).

Surviving for more than 4 weeks was positively associated with owners experiencing lifestyle limitations, owner worries about complications (eg, hypoglycemia) and the cat's QoL not being negatively affected by DM.

#### 3.4.2 Survival time

Almost two thirds of the cats (299/477, 63%) survived for more than 1 year after diagnosis, 118 (25%) survived for more than 3 years, 47 (10%) survived for more than 5 years and 7 (1.4%) survived for more than 8 years. The median survival time for cats that survived the initial 4 weeks after diagnosis but were dead at time of the questionnaire (n = 261) was 1 year (IQR 3, 0‐14 years), and for cats that were alive at the time of the questionnaire (n = 144) the median survival time was 2 years (IQR 2).

Factors remaining significant for survival time in the final linear regression model were insulin treatment, the cat's QoL and the owner's worries about medication—see Table 5. One interaction remained, between cat age at diagnosis and insulin.

**TABLE 5:** Multiple linear regression analysis of factors associated with survival time in cats with diabetes mellitus that survived more than 4 weeks after diagnosis (n = 405)

Outcome and factors	B	CI 95% (B)		*P*
Survival time				
Worries about medication vs no worries about medication	−0.64	−1.05	−0.23	.002
QoL better or same as before diagnosis vs a worsened QoL	0.60	0.16	1.05	.01
Cat age				
When treatment with insulin	−0.25	−0.33	−0.19	<.001
When no treatment with insulin	−0.03	−0.245	0.18	.77

Abbreviations: B, regression coefficient; CL, confidence interval; QoL, quality of life.

In cats that were treated with insulin (n = 360), survival time was longest in young cats, and declined by 0.25 years for every additional year of the cat's age at diagnosis. In cats that did not receive insulin (n = 45), the effect of age on survival time was not significant (*P* = .77).

Survival time increased by 0.6 years in cats with a QoL assessed as better or the same as before DM and in cats belonging to owners that did not experience worries about medication.

#### 3.4.3 Remission

Of the 405 cats that survived for more than 4 weeks, 29% (n = 118) achieved remission. Of these, 62% (n = 73) achieved remission without relapse (until the death of the cat, n = 20, or until the time of the survey, n = 53)—see Figure 1. The time from diagnosis to remission was 0 to 3 months for 22% (26/118), 4 to 6 months for 29% (34/118), 7 to 12 months for 19% (n = 22/118), and >12 months for 25% (n = 30/118).

Factors remaining significant for achieving remission in the final multiple logistic regression model were breed, type of diet and the effect of DM on the cat's QoL—see Table 4. Cats that were fed predominantly CA wet food after diagnosis had an increased chance of remission, compared to cats that were fed DM prescription diet (wet and/or dry). Norwegian Forest Cats had a higher chance of achieving remission than domestic cats. Remission was associated with a better QoL for the cat or a QoL unaffected by DM.

#### 3.4.4 Remission without relapse

The significant variables that were positively associated with remission without relapse were type of diet and the cat's QoL—see Table 4. Predominantly feeding a CA wet diet, compared to DM prescription diet (wet and/or dry), was associated with remission without relapse. Cats without relapse from remission were more likely to have a better QoL.

#### 3.4.5 Quality of life

Most owners of cats that were alive at the time of the survey (67%, 96/144) assessed their cat's overall QoL to be the same as before diagnosis. QoL was assessed to be better than before in 16% (n = 23) and worse than before diagnosis in 10% (n = 15). Almost all owners of cats that had survived for more than 4 weeks after diagnosis and were alive at the time of the survey assessed their cat's QoL at the time of the survey as excellent or good (97%, 140/144).

Most owners of cats that were dead at the time of the questionnaire assessed their cat's QoL, as affected by DM, to be worse than before diagnosis (44%, 148/333). About a third (36%, n = 121) assessed QoL as the same as before diagnosis, and 4% as better than before DM (n = 13).

The overall effect of DM on the cat's QoL is presented in Table 6.

**TABLE 6:** How diabetes mellitus affected the general quality of life for the cat according to owners, compared to before diabetes (n = 477)

				Remission			
	All cats (n = 477)	Survival ≤4 weeks (n = 72)	Survival >4 weeks (n = 405)	All (n = 118)	Relapse (n = 45)	No relapse (n = 73)	No remission (n = 287)
QoL as affected by DM							
Better^a^	36 (8%)	2 (3%)	34 (8%)	17 (14%)	3 (7%)	14 (19%)	17 (6%)
Same as before DM^a^	217 (45%)	3 (4%)	214 (53%)	79 (67%)	26 (58%)	53 (72%)	135 (47%)
Worse^b^	163 (34%)	32 (44%)	131 (32%)	14 (12%)	13 (29%)	1 (1%)	117 (41%)
Cannot assess	61 (13%)	35 (49%)	26 (6%)	0	0	0	18 (6%)

Abbreviations: DM, diabetes mellitus; QoL, quality of life.

The final multiple logistic regression model on the association between DM and the cat's general QoL included answers from owners of cats that survived for more than 4 weeks after diagnosis (n = 405). An unaffected or better QoL compared to before diagnosis was associated with both achieving remission without relapse and treatment with insulin. Expressing concern about DM limiting the life of the cat and worrying about the cat's medication were associated with an estimated QoL that was worse (Table 4).

## 4 DISCUSSION

This survey combined information on treatment, monitoring, owner perceptions and disease outcome of cats with DM. Of the cats diagnosed with DM, 85% survived for more than 4 weeks, and 63% for more than 1 year. In younger cats, a negative association was found between owner concerns about costs and survival for more than 4 weeks. Further, survival time was positively associated with a better QoL for the cat and negatively associated with owner worries about the cat's medication. The remission rate among treated cats was 29%. The chance of both achieving and staying in remission was higher for cats that were fed a CA wet diet. Remission was positively associated with a better QoL.

### 4.1 Initial survival and survival time

The initial survival rate was 85%, which is comparable to previous studies.^1^, ^25^, ^26^, ^27^ The odds of surviving for more than 4 weeks decreased slightly with increasing cat age. If the owners were worried about costs, the odds of surviving for more than 4 weeks after diagnosis were lower in younger cats, and increased with increasing cat age. This might mirror 2 things. First, the younger the cat is at diagnosis, the longer the prospective treatment will be, possibly making owners less prone to agree to treatment. Second, worries could reflect a more severe or complicated disease course among the younger cats in this study. On the contrary, the effect of age on initial survival among cats belonging to owners who experienced expectations of euthanasia was pronounced in older cats, with decreased odds of survival in cats over 15 years of age. This might be explained by less acceptance of disease negatively affecting the life of an older cat, compared to younger cats.

Survival time was positively associated with a better QoL for the cat, and was negatively associated with an owner worrying about medication. Survival time was associated with cat age in cats treated with insulin, with shorter survival time in older cats as expected.

Insulin is a general recommendation when treating DM in cats, and almost 90% of the cats surviving for more than 4 weeks after diagnosis received insulin injections. Previous research has shown that survival time is affected by the presence of concurrent diseases,^26^, ^27^ remission,^26^ and glycemic control.^27^ Here, no associations between survival time and comorbidities or achieving remission were seen. This could be partly attributable to the study design, and owners might have misinterpreted medical information about the cat. Information on glycemic control was not obtained, and the course of disease might have differed among individuals and between studied groups. Also, in the present study, cats were included with no regard to where the cat was treated, compared to studies performed at referring clinics.^26^, ^27^

In the present study, survival time was calculated as the shortest possible survival time after diagnosis, and many cats can therefore be expected to have survived for longer. There was no association between owner concerns about costs and survival time, potentially reflecting actual costs related to DM being described as having been experienced as lower than expected after a time of treatment, although still being a concern for many owners.^3^, ^29^ These results highlight the importance of informative owner communication upon DM diagnosis, discussing treatment options and the financial impact that treatment might have over a period of time.

### 4.2 Remission

The remission rate was 29%, with a 3‐fold higher chance of achieving remission for cats fed a CA wet diet (low carbohydrate, LC) compared to cats fed a veterinary prescription diet (wet and/or dry). Remission is thought to occur as a result of reversal of glucotoxicity.^21^, ^32^ By lowering and stabilizing postprandial blood glucose,^33^ facilitating the reversal of glucotoxicity, LC diets are now recommended for disease control.^23^, ^24^ Previously, reported remission rates have varied greatly. In the present study, detailed data on how remission was confirmed was not included. A presence of falsely confirmed remission cases can therefore not be excluded, and the remission rate might be exaggerated.

More than a third of the cats that achieved remission (38%) experienced relapse. There was a strong association between CA wet diet and remission without relapse, supporting the current recommendations of continuing a LC diet after remission.^22^, ^23^ An LC diet during the non‐insulin‐dependent period has previously been associated with a relapse rate of 26%,^14^ compared to relapse rates of around 30% when type of diet was not known.^34^, ^35^

The most common form of DM in cats is type 2 DM,^36^ although hypersomatotropism is attracting increased attention as a cause of insulin resistance and DM.^37^ Hypersomatotropism in cats is most likely an underdiagnosed disease,^37^ and in affected cats, successful treatment of excess growth hormone^38^—alongside traditional hypoglycemic treatment—is necessary for a chance of achieving diabetic remission. The importance of a CA wet diet to achieve remission thus indicates that many cats were type 2 diabetics, supported by the importance of diet management for treatment of T2DM in humans.^39^, ^40^

The macronutrient content of the different diets in this study was not known. The LC properties of the CA wet diet was obtained from free text answers, where a few well‐known brands of canned diet were repeatedly mentioned. The effect of a CA wet diet on remission might be caused by a high protein content,^20^, ^41^ by differing carbohydrate sources^42^ or by weight loss as a consequence of the high water content in canned foods.^43^

In the present study, Norwegian Forest Cats—a breed with an increased risk of DM^9^, ^11^, ^30^—also had increased odds of remission compared to domestic cats. To our knowledge, no previous study has reported an association between breed and disease outcome. The association between Norwegian Forest Cats and remission is interesting and requires further investigation.

There was no association between insulin treatment and remission in the present study. Previously, the associations between remission and a variety of treatment protocols, including different types of insulin, HBGM routines and diets, have been studied,^12^, ^13^, ^14^, ^15^, ^16^, ^17^, ^18^, ^19^, ^20^ with merely weak evidence of any solid associations.^21^ In the present study, 90% of the cats received insulin treatment, and about half of them (57%) practiced HBGM. The type of insulin administered, how rigorous monitoring was among owners practicing HBGM and when in relation to diagnosis insulin treatment was instituted was not known. Although most cats received insulin twice per day, no differentiation was made between cats given insulin once or twice daily upon data analysis. A suboptimal treatment protocol in a proportion of cats might have contributed to the lack of association. Also, cats with a more advanced disease course might have been treated with insulin to a higher degree, compared to cats with less progressive disease. More severely affected beta cells could have affected the chance of achieving remission in the former group. It is also possible that for some cats with a less advanced disease course, the introduction of LC diet alone is sufficient for reversal of glucotoxicity and promoting remission.

In the present study, the time until remission varied from the interval 0 to 3 months to >12 months, and no association between time until remission and maintained remission was seen. As prompt glycemic control preserves more vital beta cells,^12^, ^14^ an association between a shorter time until remission and remission without relapse would be expected. However, the time between disease onset and initiation of treatment was unknown, and no such analysis could be carried out. Also, it is somewhat surprising that a similarly large proportion of cats achieved remission more than a year after diagnosis compared to a few months after diagnosis. This might suggest that hypoglycemic treatment, although not always sufficiently instituted for early reversal of glucotoxicity, might be enough to relieve the beta cells, preserving function for a later remission. This might also reflect the role of insulin resistance in the treatment of DM in cats, where obesity and concurrent diseases have to be addressed to achieve satisfactory glycemic control. In the present study, information on how the veterinarians confirmed the DM diagnosis was not available. Inclusion of isolated cases of hyperglycemia (eg, stress hyperglycemia) can therefore not be excluded, and would influence the remission rates. However, this would have led to a high proportion of early remissions, something that was not seen.

Of the 73 cats without relapse from remission, more than two thirds (73%) were still alive at the time of the study and could still potentially relapse. Of the cats that had not achieved remission (n = 287), almost a third (27%) were alive at the time of the study, but almost all of these (97%) had received their DM diagnosis more than 2 years before the study, making remission less probable.^14^

Both achieving remission and remission without relapse were associated with a better QoL for the cat. Assessment of QoL in companion animals is complicated.^44^ In the present study, QoL was interpreted by owners, and was thus affected by their personal views. The glycemic status of the treated cats was not known. Nevertheless, the association between remission and a better QoL indicates that for an insulin‐dependent cat, the disease and its monitoring and treatment negatively affect QoL. This further emphasizes remission as a desired goal in disease management, and shows the importance of including assessment of QoL in the treatment of the diabetic cat.

### 4.3 Owners' perceptions and concerns

In the present study, around half of the owners perceived limitations in their daily life because of the cat's DM, and more than a third were concerned about complications. However, neither this perception of limitation nor worrying about complications were associated with decreased odds of the cat surviving the initial 4 weeks, that is, whether or not euthanasia was chosen in conjunction with diagnosis. A possible explanation for this is that these limitations or concerns were not experienced until after a period of treatment.

Previously recognized owner concerns include costs, limitations in owner lifestyle, worries about complications and worries about the cat's wellbeing.^2^, ^3^, ^28^, ^29^ Studies have shown that these concerns decreased with treatment time.^3^, ^29^ Positive matters include more attention being given to the cat after diagnosis^2^ and a stronger bond between the owner and the cat.^2^, ^28^, ^29^ In the present study, more than half of the owners reported a strengthened relationship with their cat.

## 5 LIMITATIONS OF THE STUDY

The main limitation of the study is that all information was obtained from owners and that a majority of the cats were not alive when their owners answered the questionnaire. This might have resulted in recall bias, as well as misinterpretations concerning the cat's medical history. In addition, detailed information about several aspects was missing. Owners who participated could represent a more motivated fraction. The definitions of diabetes mellitus and diabetic remission used in the study were formulated to fit owners, and did not conform to internationally agreed definitions,^45^ which might limit comparison with other studies. To reduce the risk of overestimating survival time, since time frames were set as the year of diagnosis and death, and not a specific date, survival time was recorded from 31 December in the year of diagnosis to 1 January in the year of death. Survival time was therefore recorded as the shortest possible time, and will be longer in reality. The present study included insured cats, and some results might differ for non‐insured cats.

## 6 CONCLUSIONS

Diabetes mellitus is a complex disease, and treatment affects the lives of both the owner and the cat. Feeding the cat a CA wet diet was associated with achieving and maintaining diabetic remission. Achieving remission is associated with a better quality of life for the cat. Therefore there is a need for further studies investigating factors associated with diabetic remission. More than 1 in 10 cats were euthanized in the first weeks following DM diagnosis. Despite this, almost two thirds of the cats (63%) survived for more than 1 year, and a quarter (25%) for more than 3 years. If the owner was worried about limitations to the cat's life and about medication, survival time was shortened. The results accentuate the association between diet and disease outcome. Also, it is crucial to recognize and manage owner distress as a part of disease management, in order to improve welfare for both owner and cat, and to improve the chances of a favorable outcome for the diabetic cat.

## CONFLICT OF INTEREST DECLARATION

Authors declare no conflict of interest.

## OFF‐LABEL ANTIMICROBIAL DECLARATION

Authors declare no off‐label use of antimicrobials.

## INSTITUTIONAL ANIMAL CARE AND USE COMMITTEE (IACUC) OR OTHER APPROVAL DECLARATION

Authors declare no IACUC or other approval was needed.

## HUMAN ETHICS APPROVAL DECLARATION

Authors declare human ethics approval was not needed for this study.

## Supporting information


**Appendix S1.** A questionnaire containing 46 questions distributed to owners of cats with diabetes mellitus containing. Data from 37 questions were analyzed in the present study. The questionnaire was translated from Swedish to English upon publication.
