# Supplementary material for: Survival, remission, and quality of life in diabetic cats
Source: J Vet Intern Med. 2023 Jan 13;37(1):58–69. doi: 10.1111/jvim.16625 (PMC9889602; doi:10.1111/jvim.16625)
Supplement: Supplementary file 1 — Appendix S1. A questionnaire containing 46 questions distributed to owners of cats with diabetes mellitus containing. Data from 37 questions were analyzed in the present study. The questionnaire was translated from Swedish to English upon publication. [file JVIM-37-58-s001.pdf]

Questionnaire used for the research article “Survival, remission and quality of life in diabetic cats”.

1. **When were you, the respondent, born?**

1929 or earlier	1970-79
1930-3911	1980-89
1940-49	1990-99
1950-5911	2000 or later
1960-69	Other/decline to answer

2. **What is your gender?**
Female
Male
Other/decline to answer

3. **What is the number of adults (18 years or older) in the household?**
1
2
3 or more
Other/decline to answer

4. **Are there any children (below 18 years of age) in the household?**
Yes
No
Other/decline to answer

5. **Where do you live?**
In a big city (more than 200 000 inhabitants)
In a town/smaller city(200-200 000 inhabitants)
In the countryside
Other/decline to answer
Comment: ____

6. **What breed is/was your cat?**
Domestic cat (including mixed breed)

Abyssinian	Norwegian Forest Cat
Bengal	Ocicat
Birman	Oriental shorthair
British Shorthair	Persian
Burmese	Ragdoll
Cornish Rex	Russian Blue
Devon Rex	Siamese
European Shorthair	Siberian (incl. Neva Masquerade)
Exotic	Somali
Maine Coon	Sphynx

Other pedigree
Other/decline to answer
Comment: ____

7. **What year was your cat born?**

1990	2003

1991	2004
1992	2005
1993	2006
1994	2007
1995	2008
1996	2009
1997	2010
1998	2011
1999	2012
2000	2013
2001	2014
2002	2015

Comment: ____

8. **What gender is/was your cat?**
   Male – neutered   Female - neutered
   Male – intact   Female – not neutered
   Other/decline to answer
   Comment: ____

9. **Is your cat alive as to this date?**
   Yes – please continue to question 12
   No

10. **What year did your cat die?**

2000 or earlier	2008
2001	2009
2002	2010
2003	2011
2004	2012
2005	2013
2006	2014
2007	2015

   Other/decline to answer
   Comment: ____

11. **What was the main cause of your cat's death**
    Trauma/accident - please continue to question 12
    High age - please continue to question 12
    Disease
    Other/decline to answer
    Comment: ____

12. **What kind of disease? *Multiple answers is possible.***
    Airway disease
    Circulatory system disease
    Diabetes or other endocrine disease
    Gastrointestinal disease

Disease in oral cavity
Orthopedic or neurological disease
Tumor disease
Urinary system disease
Other/decline to answer
Comment: ____

13. **Has your cat even been diagnosed with diabetes?** ***The diagnosis must have been made by a veterinarian. Diabetes means that the cat´s blood sugar is elevated for a longer period of time.***
Yes
No
Comment: ____

14. **What year did your cat contract diabetes?** ***Please mark the year as when you noted clear signs of diabetes. If no signs of disease were present before the diabetes diagnosis was set, please mark the year of diabetes diagnosis.***

2000 or earlier	2008
2001	2009
2002	2010
2003	2011
2004	2012
2005	2013
2006	2014
2007	2015

Other/decline to answer
Comment: ____

15. **What happened after your cat was diagnosed with diabetes?**
The cat died or was euthanized within a month after the diabetes diagnosis – please continue to question 23
The cat lived for more than a month after the diabetes diagnosis
Other/decline to answer
Comment: ____

16. **What was the reason your cat did not survive more than a month?** ***Multiple answers is possible.***
The cat did not survive despite initiated treatment
Presence of other diseases
The treatment did not work
The treatment was too difficult for me/my family
A wish to limit the suffering for the cat
The treatment was too expensive
The cat did not receive any treatment
Experienced poor support from veterinarian

17. **What treatment is your cat receiving /did your cat receive?** ***Multiple answers is possible.***
Insulin (syringe/injection) at home once a day

Insulin (syringe/injection) at home twice a day or more
Dietary adjustments
Peroral tablets for lowering blood sugar levels (e g Mindiab)
Admitted to hospital for care
No particular treatment
Other/decline to answer
Comment: ____

18. **Have you made any dietary adjustments in connection with your cat’s diabetes diagnosis?**
Yes
Yes, partly
No
Other/decline to answer
Comment: ____

19. **What diet has your cat predominantly eaten since it´s diabetes diagnosis? *If your cat have had approximately 75 % or more of one type of diet – mark this diet alternative. If your cat have had approximately 50 % of two different types of diets – mark these two diet alternatives. Canned food is denoted as wet diet.***
Veterinary diabetes prescription dry diet (e.g. Hill’s m/d, Purina DM, Royal Canin Diabetic)
Veterinary diabetes prescription wet diet (e.g. Hill’s m/d, Purina DM, Royal Canin Diabetic)
Veterinary weight loss/obesity prescription diet (e.g. Hills r/d or w/d)
Commercially available dry food
Commercially available wet food
Low carbohydrate dry food (e. g. Orijen, Carnia Lynx)
Other/decline to answer
Comment: ____

20. **Did or do you practice any glucose monitoring at home? *Multiple answers is possible.***
Yes, with blood samples
Yes, with urinary dipsticks
No
Other/decline to answer
Comment: ____

21. **Has your cat recovered from its diabetes? *Recovery meaning a normalized blood sugar and the cat no longer needing medication (insulin or oral tablets). Most cats that recover from diabetes have to continue on a special diet for the rest of their lives.***
Yes
Yes, temporary
No – please continue to question 23
Comment: ____

22. **How long did it take your cat to achieve recovery from diabetes (meaning no more medication), as from the time of diabetes diagnosis?**
0-3 months
4-6 months
7-12 months

One year or more
Other/decline to answer
Comment: ____

23. **How are/were you affected by having a cat with diabetes?** ***Multiple answers is possible.***
I feel worried about the cat’s medication
I feel worried about complications (e.g. hypoglycemia)
I think it is difficult to administer insulin
I think it is difficult to perform blood sampling
I feel worried about hurting the cat during medication or blood sampling
I perceive limitations in my life due to cat’s diabetes (e.g. difficulties travelling)
I feel worried about limitations to the cat’s life due to diabetes
I feel worried about costs
I have not been affected by my cat’s diabetes diagnosis
Decline to answer
Comment: ____

24. **How are/were you affected by having a cat with diabetes?** ***Multiple answers is possible.***
I have experienced expectations from others to start treatment
I have experienced expectations from others to euthanize
I experience increased worry that others in my life will contract diabetes
I have gained an increased awareness about diabetes
I have developed better eating habits due to my cat’s diabetes
I have developed better exercise habits due to my cat’s diabetes
I have not been affected by my cat’s diabetes diagnosis
Decline to answer
Comment: ____

25. **Have there been any conflicts in your family regarding the care of your cat?**
Yes, often
Yes, occasionally
No, we agree
No, I make the decisions myself
Other/decline to answer
Comment: ____

26. **How is/was the relationship with your cat affected by diabetes?** ***Multiple answers is possible.***
I have developed a stronger bond with my cat
My cat has developed a stronger bond with me
No change in our relationship
I have developed a weaker bond with my cat
My cat has developed a weaker bond with me (e. g. scared of injections)
Other/decline to answer
Comment: ____

27. **How has the quality of life of your cat (physical and psychical) been affected by diabetes, in general, compared to before the cat got sick? The quality of life of the cat after diabetes is:**

Better compared to before diabetes
About the same as before diabetes
Worse compared to before diabetes
I cannot assess this/decline to answer
Comment: ____

You have now answered questions concerning your cat´s diabetes. Please observe that the following questions concern the period of time BEFORE the cat contracted diabetes.

28. **How do you assess your cat´s health throughout life? *Please observe that this question concerns the time before diabetes developed.***
    Never/rarely sick or hurt – continue to question 30
    Occasionally sick or hurt – continue to question 30
    Often sick or hurt, recurrent medical problems or presence of chronic disease – continue to the next question
    Other/decline to answer – continue to the next question
    Comment: ____

29. **If often sick or hurt/recurrent medical problems/chronic disease, in what way was your cat affected by this/these medical issues? *Multiple answers is possible.***
    Airway disease
    Circulatory system disease
    Endocrine disease
    Gastrointestinal disease
    Infectious disease
    Disease in oral cavity
    Orthopedic or neurological disease
    Disease in reproductive system
    Skin disease
    Tumor disease
    Urinary system disease
    Other/decline to answer
    Comment: ____

30. **Is or was your cat vaccinated?**
    Yes, yearly
    Yes, every other or third year
    Yes, as a kitten
    No, my cat is not vaccinated
    Don´t know/decline to answer
    Comment: ____

31. **Has your cat received any of the following medications during the last year? *Please observe that for the diabetic cat, this question concerns the year before diabetes developed. Multiple answers is possible.***
    Yes, contraceptives (e. g. Promon, Perlutex)
    Yes, injectable contraceptives (e. g. Promon)

Prednisolone peroral tablets (e. g. Prednisolone, Medrol)
Injectable prednisolone (e. g. Depo-Medrol)
No, the cat has not received any of the above mentioned medications
Don´t know/other
Comment: ____

32. **Describe the diet of your cat.** ***Please observe that for the diabetic cat, this question concerns the year before diabetes developed. If your cat had approximately 75 % or more of one type of diet – mark this diet alternative. If your cat had approximately 50 % of two different types of diets – mark these two diet alternatives. If the given alternative does not fit your cat – mark the "Other/don´t know" alternative.***
Dry food
Canned/wet food
Home cooked food, BARF/raw diet
Food scraps
Mice and other prey
Other/don´t know
Comment: ____

33. **Approximately how often did you feed your cat?** ***Please observe that for the diabetic cat, this question concerns the year before diabetes developed. If you e. g. serve wet food twice a day but offer free access of dry food – please mark the "free feeding" alternative.***
Once a day
Twice a day
Three times a day
Free feeding
Other/don´t know
Comment: ____

34. **Describe the feeding habits of your cat.** ***Please observe that for the diabetic cat, this question concerns the year before diabetes developed.***
Greedy eater/finished meal immediately
Finished meal within a few hours
Snacking/many small meals throughout the day
Picky eater/frequently leaves food
Other/don´t know
Comment: ____

35. **Describe the body condition of your cat.** ***Please observe that for the diabetic cat, this question concerns the year before diabetes developed.***
Overweight
Normal weight
Underweight
Other/don´t know
Comment: ____

36. **Describe the body condition of your cat, using the following template.** ***Please observe that for the diabetic cat, this question concerns the year before diabetes developed.***

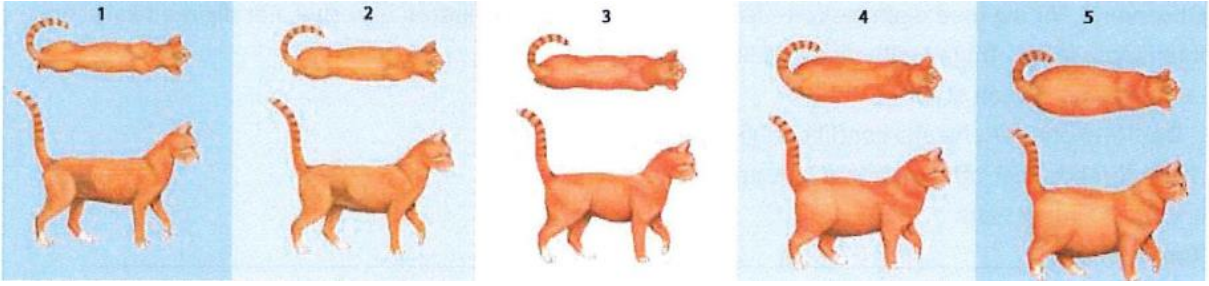

Category explanation:
1 No fat can be felt over the ribs, hollow waist
2 The ribs are easy palpable with a thin fat layer on top, distinctly visible waist
3 The ribs can be palpated with a thin fat layer on top, visible waist
4 Difficult to palpate ribs, no visible waist, pendulous abdomen
5 A thick layer of fat on top of ribs, pendulous abdomen

1
2
3
4
5
Other/decline to answer
Comment: ____

37. **Have you tried to make your cat lose weight? *Please observe that for the diabetic cat, this question concerns the year before diabetes developed.***
Yes
No
Other/decline to answer
Comment: ____

38. **How many adult cats (>6 months of age) were there in the household, in total? *Please observe that for the diabetic cat, this question concerns the year before diabetes developed.***
1
2-3
4-8
9 or more
Other/decline to answer
Comment: ____

39. **If your cat lives together with other cats, how did the cat group function? *Please observe that for the diabetic cat, this question concerns the year before diabetes developed.***
The cat group was in harmony
There was occasional conflicts in the cat group
There were frequent conflicts in the cat group
Other/decline to answer
Comment: ____

40. **Were there any other animals in the household? *Please observe that for the diabetic cat, this question concerns the year before diabetes developed. Multiple answers is possible.***
Yes, dog
Yes, smaller animals (e. g. rabbits, guinea pigs, small birds)
Yes, animals in aquarium/terrarium

No, only cat
Other/decline to answer
Comment: ____

41. **Were there any large changes in your cat´s life during the last year before diabetes developed?** ***Large change meaning relocation, new family member (e. g. baby or other animal), bigger renovating projects, loss of an animal friend or other.***
Yes
No
Other/decline to answer
Comment: ____

42. **Was your cat an indoor or an outdoor cat?** ***Please observe that for the diabetic cat, this question concerns the year before diabetes developed.***
Indoor only
Indoor with access to balcony/play pen/leash walks
Indoor with access to outdoors for part of the year (e. g. summer time, at the country house)
Outdoor (both outdoor and indoor)
Outdoor only
Other/decline to answer
Comment: ____

43. **Describe your cat´s degree of physical activity.** ***Please observe that for the diabetic cat, this question concerns the year before diabetes developed. Also, please observe that it is considered normal cat behavior to sleep or rest for an average of 16 hours a day.***
Very active (plays, hunts, climbs for many times a day)
Normal degree of activity (plays/hunts every day)
Inactive (seldom or never plays or hunts, mostly sleeps or rests)
Other/decline to answer
Comment: ____

44. **Please describe your attitude towards your cat.**
My cat is everything to me
My cat means a lot to me
My cat is quite important to me
My cat is not that important to me

45. **How do you assess your cat's quality of life today?** ***Please observe that for cats that are no longer alive, the question concerns the cat´s last time in life.***
Excellent
Good
Less good
Poor
Other/decline to answer
Comment: ____

46. **How do you assess your cat's quality of life as throughout life, in general?**
Excellent

Good
Less good
Poor
Other/decline to answer
Comment: ____

Other comments: ____
